# Perceived viewing-experience enhancement and audience acceptance: applying TAM to AI-based sports broadcasting services

**DOI:** 10.3389/fspor.2026.1945033

**Published:** 2026-10-16

**Authors:** Liuxin Li

**Affiliations:** Independent Researcher, Guangzhou, Guangdong, China

**Keywords:** artificial intelligence, behavioral intentions, perceived viewing-experience enhancement, sports broadcasting services, technology acceptance model (TAM)

## Abstract

**Introduction:**

Despite the rapid proliferation of artificial intelligence (AI) technologies in sports broadcasting, empirical evidence validating the Technology Acceptance Model (TAM) and experiential outcomes in this specific context remains limited. This study aims to address this gap by investigating the statistical relationships among core TAM constructs, usage attitude, behavioral intention, and perceived viewing-experience enhancement within AI-based sports broadcasting services.

**Methods:**

Based on the theoretical framework of TAM, this study collected valid data from 630 Chinese respondents. Structural equation modeling (SEM) was employed to quantitatively test the hypothesized research model and examine the interrelationships between the proposed variables.

**Results:**

The empirical results demonstrate that perceived ease of use positively predicts perceived usefulness, and both perceived ease of use and perceived usefulness exert significant positive effects on users' usage attitude. Furthermore, usage attitude is positively associated with behavioral intention and perceived viewing-experience enhancement. Notably, usage attitude plays a full mediating role in the relationships between the two perceptual antecedents (perceived ease of use, perceived usefulness) and the two outcome variables (behavioral intention, viewing-experience enhancement).

**Discussion:**

This research contributes context-specific empirical validation to the TAM framework in the emerging domain of AI-driven sports broadcasting. It extends existing TAM literature by integrating individual-level experiential outcomes alongside traditional behavioral intention indicators in sports media consumption. Practically, the findings provide actionable insights for optimizing user experience and improving service design for AI sports broadcasting platforms.

## 1 Introduction

The global sports media industry is experiencing far-reaching digital transformation fueled by the rapid integration of artificial intelligence. Functions such as real-time AI-generated highlights, intelligent automated commentary, personalized content recommendations, and immersive viewing modes are reshaping how audiences engage with and consume live sports broadcasts. This transition is particularly marked in the Chinese context. Evolving from esports toward multifaceted virtual events, China's digital-sports landscape exemplifies the broader course of technological innovation amid concurrent opportunities and challenges (1).

Artificial intelligence has gained growing traction across multiple dimensions of modern sports broadcasting. Beyond backend technical support such as intelligent video surveillance for large-scale tournaments (2), generative AI is creating new front-end audience-oriented values: it facilitates content production, delivers tailored recommendations, and supports high-quality circulation of sports-related cultural content (3, 4). Complementary digital-driven approaches, including digital-twin applications and omni-media communication frameworks, further accelerate the digital upgrade of the broader sports industry (5–7). On the international stage, the Olympic Agenda for Artificial Intelligence charts the global trajectory of AI-enabled sport development and carries meaningful implications for Chinese sports-media strategies (8). The Paris Olympics further illustrated AI's capacity to elevate audiovisual sports communication through collaborative technological empowerment (9). Collectively, these developments underscore AI's transformative potential for professional sports operations, event presentation, and multi-stakeholder governance of online sports events (10, 11).

While numerous existing studies examine regulatory frameworks, developmental trajectories, value potentials, and practical challenges of AI within sports events from supply-side and policy-oriented standpoints, demand-driven empirical evidence concerning audience responses remains limited. Much prior work adopts macro-level or technology-centered lenses, with relatively little attention paid to audience-level psychological and experiential responses. One recent study explored Chinese audiences' readiness for AI-infused sport communication from the perspective of media affordances, noting both disruptive potentials and associated downsides (12). Even so, few studies draw on well-validated technology-acceptance frameworks to investigate how core acceptance perceptions correlate with subjectively experienced viewing outcomes among audiences of AI-based sports broadcasting services.

Within sport-media research, empirical applications of the Technology Acceptance Model predominantly focus on behavioral intention, a canonical outcome construct originally embedded in TAM's theoretical system (13). Prior TAM-informed sport-streaming research has advanced our understanding of user adoption, yet it largely stops at predicting usage and payment intention, with limited attention to subjective experiential outcomes that reflect audiences' actual viewing gains after engaging with technology-enhanced broadcast functions. Although media-effects scholarship has long highlighted cognitive, affective, and immersive viewing responses, such experiential evaluations are rarely incorporated into TAM-based analytical frameworks for AI sports-broadcasting contexts. This study attempts to introduce the concept of enhanced perceived viewing experience into the theoretical network of TAM as an outcome variable at the individual level. This construct captures subjective audience evaluations including game comprehension, emotional immersion, interactivity, and viewing satisfaction, rather than macro-scale mass-media diffusion or event reach. The underlying theoretical logic follows: audiences' cognitive appraisals of technology shape subjective usage attitudes, which in turn are expected to correlate with both the established TAM outcome of behavioral intention and this experiential viewing-related outcome.

Building on this empirical and theoretical background, the present study adopts a technology-acceptance perspective to examine audience acceptance correlates for AI-based sports broadcasting services. It investigates how perceived usefulness and perceived ease of use predict usage attitudes, and further how these attitudes associate with perceived viewing-experience enhancement and behavioral intention, filling the above-identified empirical gap.

The specific research questions guiding this work are as follows:

RQ1: How do perceived usefulness and perceived ease of use predict audience attitudes toward AI-based sports broadcasting services?
RQ2: To what extent are audience usage attitudes associated with behavioral intention and perceived viewing-experience enhancement?
RQ3: Do usage attitudes mediate the associations between technology-related perceptual predictors and the two outcome variables?

Theoretically, this study offers a context-specific adaptation of the established TAM framework for AI-based sports broadcasting services. It supplements existing TAM applications in sport-media domains by simultaneously accounting for both behavioral willingness and subjectively perceived viewing-experience outcomes. Testing these associational pathways among Chinese sport-media audiences offers further empirical evidence regarding TAM's applicability within non-Western digital-sport environments.

Practically, the findings can offer actionable reference for sports broadcasters, media platforms, and AI service developers. Insights may inform product-oriented iterations centered on measurable user-experience dimensions: optimizing perceived ease-of-use of AI broadcast interfaces, strengthening functional usefulness of AI-enabled features, delivering personalized content, and fostering positive user attitudes toward AI-supported viewing functions.

## 2 Theoretical framework

### 2.1 Technology acceptance model and its extensions

The Technology Acceptance Model (TAM) originated in the fields of ecological psychology, information systems research, and human-computer interaction, and was formally proposed by Davis in 1989. Based on Fishbein and Ajzen's Theory of Reasoned Action (TRA), this model focuses on the dynamic interaction between technological characteristics, user perceptions, and usage behavior (14). Within the domain of sports-event communication, the Technology Acceptance Model offers a robust theoretical foundation for understanding audience perceptions and acceptance of AI-based sports broadcasting services. It helps unpack how technological attributes of these AI-powered broadcasting features correlate with audience acceptance intentions, continued-usage behavioral tendencies, and subjectively perceived viewing-experience enhancement.

The core logic of the Technology Acceptance Model (TAM) posits that user acceptance of new technologies is primarily associated with two central perceptual beliefs: Perceived Usefulness (PU) and Perceived Ease of Use (PEOU). These two constructs relate to behavioral intentions (BI) either directly or via usage attitude (UA), which in turn serves as a predictor of real-world usage behavior. Drawing on the core logic of the Theory of Reasoned Action (TRA), TAM adapts attitudinal-formation mechanisms for information-technology contexts and has emerged as one of the most influential frameworks within information-systems research. A large body of empirical work and bibliometric reviews has supported TAM's robustness across diverse settings. One bibliometric analysis covering 2,399 TAM-related publications spanning 2010–2020 demonstrates that TAM retains substantial explanatory capacity for emerging-technology domains including artificial intelligence, educational technology, and digital media (15).

The theoretical extensions of TAM have broadened its scope of applicability. Venkatesh and Davis (2000) developed TAM2, which incorporated external predictors including subjective norms and image to account for social-related contextual factors within technology acceptance processes. Subsequently, Venkatesh et al. synthesized eight theoretical frameworks—including TRA, TAM, and TPB—to formulate the Unified Theory of Acceptance and Use of Technology (UTAUT). UTAUT specifies four core constructs: performance expectancy, effort expectancy, social influence, and facilitating conditions, alongside moderating variables of gender, age, experience, and voluntariness. This integrated framework yields enhanced predictive capacity for complex emerging-technology contexts and exhibits strong explanatory performance in both organizational and consumer-user settings (16). In 2008, Venkatesh and Bala put forward TAM3, which further refined the set of antecedent predictors shaping perceived ease of use.

In sports-media communication scholarship, the Technology Acceptance Model has been frequently adopted to investigate payment motivations for online sports live streaming, sports-app adoption, wearable-device utilization, and consumption of AI-generated sport content (17). One related study analyzed user content-consumption behaviors on OTT sports platforms and proposed a sport-specific adaptation of TAM, examining associations between sport-related motivation, context-aware features, and user acceptance outcomes (18). While these existing works lay a valuable empirical foundation for understanding technology-user dynamics within China's fast-growing digital-sports ecosystem, most of them center on behavioral-acceptance indicators and give limited consideration to subjectively perceived viewing-experience outcomes tied specifically to AI-based sports broadcasting services.

### 2.2 Antecedent variables: perceived usefulness and perceived ease of use

Perceived usefulness, as defined by Davis, refers to the extent to which an individual believes using a given technological system can improve task-related performance and subjective user experience. Within the context of AI-based sports broadcasting services, perceived usefulness manifests through experiential and informational gains brought by AI functions. For instance, AI-generated real-time event highlights reduce users' manual searching and filtering costs and boost viewing efficiency; personalized recommendation algorithms deliver event content aligned with individual viewing preferences; intelligent commentary supplements real-time match statistics and tactical interpretations to enrich audiences' information acquisition. Prior scholarship identifies perceived usefulness as a central perceptual construct linked to users' attitudinal responses toward technology. When audiences perceive tangible experiential benefits from AI-based sports broadcasting services, they tend to form more favorable evaluations and show greater willingness to continue using such services.

Perceived ease of use captures the degree to which an individual believes that operating a technological system requires minimal time and cognitive effort (13). For AI-based sports broadcasting services, this construct is reflected in intuitive platform interfaces, natural human-computer interaction, low learning requirements, and smooth operational workflows. Even feature-rich AI broadcasting functions may encounter user resistance if operation procedures are overly complex and impose heavy cognitive burdens. Within the canonical TAM framework, perceived ease of use is expected to correlate not only with usage attitudes directly but also with perceived usefulness: reduced operational friction can enhance the perceived functional value of technology, serving as a fundamental precondition for user initial acceptance.

Meta-analytic TAM reviews have documented consistent associational patterns between perceived ease of use, perceived usefulness, and usage attitudes across consumer-technology contexts (19). Research focused on sports-media settings further suggests that perceived ease of use may relate to overall technology acceptance partly via its association with perceived usefulness, which resonates with the viewing scenario examined in the present study (18). Drawing on this theoretical and empirical foundation for the AI-based sports broadcasting context, the following hypotheses are proposed:

H1: Perceived Ease of Use is positively associated with Perceived Usefulness.
H2: Perceived Usefulness is positively associated with Usage Attitude.
H3: Perceived Ease of Use is positively associated with Usage Attitude.

### 2.3 Usage attitude as a direct predictor of outcome constructs

Usage attitude describes users' holistic subjective evaluations, encompassing positive or negative appraisals toward a given technology (13). As a core psychological construct within the Technology Acceptance Model, usage attitude is predictive of user-level technology-related responses. Critical meta-analytic work on technology-acceptance frameworks highlights that user attitudes constitute a vital psychological bridge linking pre-existing technology perceptions to subsequent user outcomes (20).

Behavioral intention constitutes a core outcome construct within technology-acceptance research. It describes individuals' subjective willingness and planned future actions to adopt, utilize, and engage with given technologies and related services, and serves as a key predictor linking user cognition to prospective usage behavior (13, 16). Within the context of AI-based sports broadcasting services, behavioral intention covers multiple user tendencies: willingness to continue watching AI-enhanced broadcast content, sharing AI-generated highlight clips, participating in interactive event discussions, and subscribing to premium AI-supported viewing services. As a central indicator of user technology acceptance, it is also relevant to platform-oriented outcomes such as audience engagement, user loyalty, and word-of-mouth engagement (21).

In addition to behavioral intention, this model introduces perceived viewing-experience enhancement (PVE) as a second outcome variable. Operationalized at the individual-user level, this construct captures audiences' subjective evaluations, including improved match comprehension, emotional immersion, perceived interactivity, and viewing satisfaction. Rooted in the nomological network of the Technology Acceptance Model, PVE is conceptualized as a further user-centric experiential outcome alongside the well-established construct of behavioral intention. Importantly, the inclusion of PVE is not intended merely as an additional correlated outcome variable. Classic TAM primarily explains technology adoption through the prediction of behavioral intention in predominantly utilitarian or task-oriented settings. In the present context of AI-based sports broadcasting, which represents an inherently hedonic and experience centered form of media consumption, the core value that audiences seek is the enhancement of the viewing experience itself. By positioning PVE as a parallel endogenous construct alongside behavioral intention, the model extends TAM's explanatory scope from “whether and why audiences intend to use the technology” to “how technology related perceptions translate into subjectively experienced gains in match comprehension, emotional immersion, interactivity, and overall viewing quality.” This dual outcome specification therefore addresses a theoretical limitation of standard TAM applications in experiential media domains: the relative neglect of individual level experiential consequences that constitute the primary purpose of consumption. In this sense, PVE functions as a theoretically motivated extension that enables the framework to account for both adoption willingness and experiential outcomes within a single nomological network.

Empirical evidence across diverse digital-media settings has documented that favorable user attitudes are associated with higher behavioral willingness to adopt, continue using, and share technology-enabled content (22–25). Nevertheless, most existing work focuses narrowly on behavioral-sharing or usage-intention indicators. Less attention has been devoted to how attitude corresponds to subjectively perceived experiential gains for audiences consuming AI-enhanced sport-media content.

Within the context of AI-based sports broadcasting services, favorable usage attitudes reflect audiences' positive subjective judgements of AI-enabled viewing features. Theoretical reasoning suggests that favorable usage attitudes are expected to be positively associated with both outcome constructs. Audiences holding positive attitudes toward AI-based broadcasting services tend to report greater willingness for continued consumption and engagement. Meanwhile, positive attitudinal evaluations also correspond to higher self-reported PVE, as audiences with favorable appraisals are more likely to perceive improved experiential gains from AI-supported broadcast functions. Drawing on the above theoretical reasoning tailored to AI-based sports broadcasting services, the following hypotheses are proposed:

H4: Usage Attitude is positively associated with Behavioral Intention.
H5: Usage Attitude is positively associated with perceived viewing-experience enhancement.

### 2.4 The mediating role of usage attitude

Within the context of AI-based sports broadcasting services, usage attitude represents users’ holistic psychological evaluation formed after weighing the perceived merits and potential risks of AI-driven viewing functions. On the positive side, desirable attributes including improved viewing efficiency, personalized content delivery, and multifaceted event interpretation may contribute to favorable usage attitudes. Conversely, concerns such as insufficient content accuracy, privacy-security risks, and limited human-like qualities in human-computer interaction may give rise to negative user perceptions. From a theoretical standpoint, audiences' perceptual beliefs about AI broadcasting features are expected to relate to subsequent behavioral intention and perceived viewing-experience enhancement predominantly through the shaping of usage attitude.

Classic technology-acceptance scholarship suggests that associations between perceived usefulness, perceived ease of use and user-level outcomes are seldom fully direct; instead, these antecedents operate largely indirectly via the psychological mediator of usage attitude. Early extensions of TAM illustrated that user technology perceptions do not on their own determine final user-related outcomes, with usage attitude functioning as a key psychological linkage connecting individual cognition to subsequent user responses (26). Meta-analytic syntheses drawing on TAM and UTAUT further support this view: usage attitude bridges perceptual antecedents and downstream user-centered outcomes and constitutes a core mechanism for understanding user technology adoption (27).

This attitudinal-mediation pattern has received empirical support across a broad range of digital-technology and media contexts (28–34). Although these prior works cover educational applications, immersive media, social-network platforms and human-machine collaborative AI systems, few have examined such mediating pathways specifically for audiences of AI-based sports broadcasting services. Drawing on this body of cross-context evidence, the present study adopts an a-priori full-mediation theoretical specification: perceived ease of use and perceived usefulness are expected to relate to behavioral intention and perceived viewing-experience enhancement entirely via usage attitude, without hypothesized direct paths from perceptual predictors to the two outcome constructs.

Against this theoretical background tailored to AI-based sports broadcasting services, the following hypotheses are proposed:

H6a: Usage Attitude mediates the association between Perceived Ease of Use and Behavioral Intention.
H6b: Usage Attitude mediates the association between Perceived Usefulness and Behavioral Intention.
H7a: Usage Attitude mediates the association between Perceived Ease of Use and perceived viewing-experience enhancement.
H7b: Usage Attitude mediates the association between Perceived Usefulness and perceived viewing-experience enhancement.

### 2.5 Research model

Figure 1 displays the theoretical model of this study. Grounded in the canonical nomological framework of the Technology Acceptance Model (TAM), which inherently takes behavioral intention as a core endogenous outcome, this study further introduces perceived viewing-experience enhancement as a supplementary user-centered outcome variable. Within the context of AI-based sports broadcasting services, the established model treats perceived viewing-experience enhancement and behavioral intention as two parallel, equally important endogenous constructs. It systematically elucidates the associational pathways through which AI-related technological perceptions shape audience behavioral intentions and subjective viewing experiences via usage attitude-based direct and full-mediation mechanisms.

**Figure 1:**
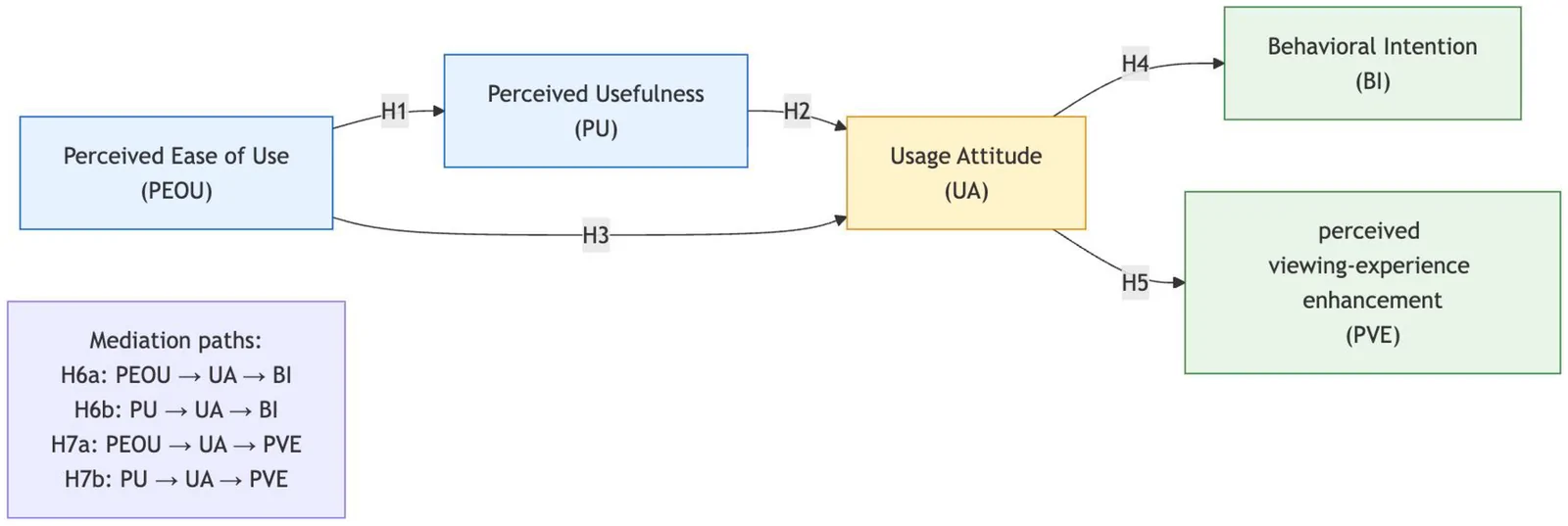
Theoretical model framework.

## 3 Methodology

### 3.1 Data collection

This study conducted anonymous questionnaire-based data collection from March to April 2026. The questionnaire was disseminated via online media platforms through calls for voluntary participants. Using convenience sampling and snowball sampling, invitations were posted on online media outlets to recruit eligible voluntary respondents, and the survey link was also distributed to personal acquaintances who met the eligibility criteria. These acquaintances further forwarded the questionnaire to other qualified potential participants. No dedicated fixed commercial recruitment panel was utilised. All investigation procedures strictly adhered to privacy protection principles. Specifically, the questionnaire was distributed anonymously via the Wenjuanxing online platform without collecting any personally identifiable information, including names, phone numbers, IP records, location data, or other sensitive content. No participant tracking, data tracing, or personal data storage was involved throughout the research process.

A mandatory screening question was placed at the very start of the questionnaire. Respondents were required to confirm that they had actual hands-on usage experience with AI-enhanced sports-broadcasting functions; only respondents providing positive confirmation could proceed to the main survey module. This screening rule excluded participants who only had superficial exposure to AI-related sports content without practical usage experience.

The initial dataset contained 792 submitted questionnaires. Multi-stage data-cleaning procedures were implemented sequentially. First, duplicate submissions were identified: responses with completely identical answers across all questionnaire items were marked as duplicate submissions, resulting in the removal of 41 responses. Second, careless and logically invalid responses were eliminated, including straight-lining, logically contradictory replies, and submissions with unreasonably short completion duration, removing an additional 95 responses. After these two quality-control steps, 656 responses remained. Third, responses from participants under 18 years of age were excluded for ethical considerations, removing 26 responses. After completing all cleaning steps, a final analytic sample of 630 valid adult responses was retained for subsequent empirical analysis.

According to demographic data (Table 1), 37.3% of respondents were female and 62.6% were male. Regarding age, the 18–24 age group comprised the largest proportion, reaching 52.5% of the total sample. This was followed by the 25–34 age group, accounting for 20.4%. In terms of educational background, bachelor degree (63.1%) was the most common, followed by junior college degree (16%).

**Table 1:** Demographic statistics*.*

Dimension	Grouping	Sample size	percentage
gender	Male	395	62.60%
	Female	235	37.30%
age group	18–24 years old	331	52.50%
	25–34 years old	129	20.40%
	35–44 years old	72	11.40%
	45 years and older	98	15.50%
Education	High school and below	52	8.20%
	Junior college	101	16%
	Bachelor	398	63.10%
	Master's degree or above	79	12.50%
Weekly sports viewing time	I hardly ever watch	69	10.90%
	Less than 2 h	221	35%
	2–5 h	206	32.60%
	5–10 h	78	12.30%
	10 h or more	56	8.80%

### 3.2 Measurement

All multi-item scales were measured on a five-point Likert scale ranging from 1 (strongly disagree) to 5 (strongly agree). Demographic variables were included as control variables.

The measurement items were adapted from well-established scales in the technology acceptance and media effects literature and contextualized to AI-based sports broadcasting services. The original English items were translated into Chinese and subsequently back-translated by the research team to ensure conceptual equivalence. Minor wording adjustments were made to improve clarity and contextual relevance for Chinese respondents. Although a formal external expert panel and large-scale pilot test were not conducted, the final items demonstrated satisfactory reliability and validity in the main study (detailed results are reported in the Results section).

The sources, number of final items, and representative final wording for each construct are presented in Table 2.

**Table 2:** Measurement constructs, sources, and final items*.*

Construct	Original Source(s)	No. of Final Items	Adaptation Approach	Final Items (Representative Wording)
Perceived Ease of Use	Davis (1989); Venkatesh & Davis (2000)	4	Translated, back-translated, and contextualized to AI sports broadcasting features	(a) I can easily learn how to use AI-based sports viewing features(e.g., AI highlight replay, multi-angle viewing). (b) The interface of AI sports broadcasting platforms or features is easy for me to operate. (c) I find it easy to use features such as AI intelligent commentary or personalized recommendations. (d) (d)Overall, I find AI-based sports broadcasting services easy to use.
Perceived Usefulness	Davis(1989); Venkatesh & Davis(2000); Sun et al. (2021)	4	Translated, back-translated, and adapted to the information and viewing benefits of AI features	(a) Using AI-based sports broadcasting features helps me watch games more effectively. (b) AI-recommended content based on my interests is useful for my sports viewing. (c) AI intelligent commentary helps me obtain relevant information more efficiently. (d) Overall, AI-based sports broadcasting services are useful for my sports viewing experience.
Usage Attitude	Davis (1989); Ajzen (1991)	4	Translated, back-translated, and modified to reflect affective evaluation of AI sports broadcasting	(a) I enjoy watching sports events through AI-based sports broadcasting services. (b) Using AI-based sports broadcasting services is a good idea. (c) I have a positive attitude toward AI-based sports broadcasting services. (d) Overall, I like using AI-based sports broadcasting services.
Perceived Viewing-Experience Enhancement (PVE)	McQuail (2010); Achen et al. (2017); Li et al. (2026)	4	Translated, back-translated, and reconceptualized as individual-level perceived enhancement of viewing experience	(a) AI-based sports broadcasting services helped me gain a deeper understanding of the games. (b) AI-based sports broadcasting services enhanced my emotional engagement while watching the games. (c) AI-based sports broadcasting services improved the interactivity and immersion of my viewing experience. (d) (d)Overall, AI-based sports broadcasting services improved the quality of my sports viewing experience.
Behavioral Intention (BI)	Davis (1989); Venkatesh et al. (2003); Sun et al. (2021)	4	Translated, back-translated, and contextualized to continued use and recommendation intentions	(a) I am willing to continue using AI-based sports broadcasting services in the future. (b) I will recommend AI-based sports broadcasting services to my friends or family. (c) I plan to increase the frequency of using AI-based sports broadcasting services. (d) If more AI-based sports broadcasting services become available, I will actively choose to use them.

Although the four PVE items tap distinct experiential sub-facets including cognitive comprehension, affective engagement, interactivity-immersion and global viewing quality, this study treats PVE as a unidimensional reflective first-order construct. Theoretically, all four indicators are conceived as manifestations of a unified higher-order subjective evaluation of AI-enhanced sports-broadcasting consumption: they represent different observable manifestations of the same underlying latent perceived-experience enhancement rather than conceptually separate exogenous constructs (35, 36). While cognitive, affective and immersive responses can be distinguished conceptually, in the sports-media-audience context these experiential responses are expected to co-occur and covary closely for a given viewer, justifying a reflective unidimensional operationalization. To empirically examine this modelling choice, a competing measurement-model comparison was performed (see Results section).

### 3.3 Analytical methods

Prior to hypothesis testing, data pre-processing and preliminary descriptive assessments were carried out for the survey dataset. Internal consistency reliability of each latent construct was evaluated using Cronbach's *α* coefficient. Confirmatory factor analysis (CFA) was employed to validate the measurement model, where model fit, convergent validity (assessed via composite reliability and average variance extracted), and discriminant validity were examined. Discriminant validity was double-checked with both the Fornell-Larcker criterion and the heterotrait-monotrait (HTMT) ratio. Potential common method bias originating from self-report questionnaires was evaluated through two complementary approaches: Harman's single factor test and a latent common method factor (CMF) technique. For the latent common method factor approach, all survey items loaded onto both their respective substantive latent construct and an additional common method latent factor. Global model fit indices between baseline five factor CFA and CMF augmented model were formally compared. Moreover, standardized substantive indicator loadings from the two models were contrasted to quantify material shifts in indicator construct relationships. Furthermore, to validate the unidimensional specification for perceived viewing-experience enhancement (PVE), a competing confirmatory-factor-analysis model was estimated. In this alternative specification, the four PVE items were separated into four distinct latent factors (cognitive comprehension, affective engagement, interactivity-immersion, global viewing quality), rather than loading onto a single unified PVE construct. Model fit indices and chi-square difference test were used to compare the unidimensional baseline against this multi-factor competing model.

Structural equation modelling (SEM) was subsequently utilised to test the proposed research hypotheses. Guided by theoretical expectations, a full-mediation baseline model was specified, such that perceived ease of use and perceived usefulness had no direct predictive paths to behavioral intention and perceived viewing-experience enhancement. Drawing on sport-media consumption and technology-acceptance research, four demographic variables were included as control variables for theoretical reasons. Gender has been shown to shape preferences and evaluative responses toward digital sports-media services (37). Age is associated with differences in digital literacy and openness to emerging AI-powered media functions, thereby influencing audience attitudes and experiential assessments (12). Educational background often corresponds to divergent information-processing patterns when encountering technology-enhanced sport content. Weekly sports-viewing duration serves as a behavioral indicator of sport-media involvement; audiences with higher habitual viewing involvement tend to form different attitudinal, behavioral, and experiential evaluations of AI-based sports-broadcasting services (12). Demographic variables including gender, age, education level, and weekly sports-viewing duration were therefore entered as controls. To assess whether substantive conclusions hinge on these demographic covariates, an alternative full-mediation structural model excluding all four controls was estimated for robustness comparison. To empirically justify this full-mediation specification, a competing partial-mediation nested model was built, and model comparisons were performed using the chi-square difference test alongside AIC and BIC information criteria. The mediating roles of attitude towards use were estimated through 5,000 bootstrap resamples; bias-corrected accelerated BCa 95% confidence intervals were reported to determine the significance of indirect effects. All statistical analyses were implemented in R, drawing primarily on the lavaan and psych packages.

## 4 Results

This study included five latent variables in the measurement model: perceived ease of use, perceived usefulness, usage attitude, perceived viewing-experience enhancement, and behavioral intention. Analyses were conducted based on 630 valid responses with no missing values. Cronbach's *α* coefficients demonstrated satisfactory internal consistency for all constructs, with each *α* value exceeding 0.7 and an overall scale *α* of 0.921 (Table 3). The confirmatory factor analysis showed that the measurement model achieved acceptable fit: $\chi^{2}/df\;=2.31$, CFI = .942, TLI = .935, RMSEA = .046, SRMR = .038. All constructs exhibited adequate convergent validity, as AVE values were greater than 0.50 and CR values exceeded 0.70 (Table 4). For discriminant validity, the Fornell–Larcker criterion was satisfied, with the square root of the AVE for each construct larger than its correlations with other latent variables (Table 5). The HTMT ratios for all pairs of constructs were below the strict cutoff of 0.85 (Table 6), further confirming discriminant validity with no severe conceptual overlap. To verify the unidimensional treatment of PVE, a competing multi-factor measurement model was tested, in which the four PVE items were assigned to four separate latent experiential factors. Model comparisons are presented in Table 7. The unidimensional PVE baseline model yielded substantially better fit: $\Delta \chi^{2\;}=127.41$, Δdf=6,(p<.001). These empirical results support treating PVE as a single first-order latent construct in the present dataset. Standardized factor loadings for each PVE indicator were 0.761, 0.784, 0.792, 0.813 respectively, all >0.70 and statistically significant (p<.001), demonstrating that each item adequately reflects the underlying PVE latent variable. It is noteworthy that the zero-order bivariate correlation between composite PVE sum-score and behavioral intention reached (r=.853), whereas the latent-variable correlation derived from CFA was (r=.712). This reduction is methodologically expected: the raw observed-variable correlation is contaminated by random measurement error; latent-construct correlations from CFA statistically correct for measurement-attenuation, yielding more conservative disattenuated associations (38). Common method bias diagnostics are summarised in Supplementary Appendix Table S2-1. Harman's unrotated single factor test showed the largest factor explained 38.72% of total variance, well below the conventional 50% threshold. Comparing baseline five factor CFA against the latent common method factor augmented model: introducing the common method factor produced only marginal global fit improvements (CFI increased by 0.006; RMSEA decreased by 0.003). Inspection of standardized substantive indicator loadings (Supplementary Appendix Table S2-2) revealed only minor magnitude shifts: mean absolute loading change = 0.031; maximum absolute change = 0.078. No large magnitude indicator loading alteration or reversal of substantive indicator construct associations was detected. Collectively, these diagnostics suggest common method bias does not constitute a serious threat to the present empirical findings.

**Table 3:** Scale reliability analysis*.*

Variables	Number of questions	Cronbach's *α*
perceived ease of use	4	0.852
perceived usefulness	4	0.867
usage attitude	4	0.879
perceived viewing-experience enhancement	4	0.883
behavioral intention	4	0.891
Full scale	20	0.921

**Table 4:** Convergent validity analysis*.*

Variables	AVE	CR
perceived ease of use	0.592	0.853
perceived usefulness	0.615	0.868
usage attitude	0.632	0.879
perceived viewing-experience enhancement	0.641	0.883
behavioral intention	0.655	0.891

**Table 5:** Fornell-larcker discriminant validit*y.*

Variables	PEOU	PU	UA	PVE	BI
PEOU	0.769				
PU	0.623	0.784			
UA	0.589	0.721	0.795		
PVE	0.521	0.658	0.703	0.801	
BI	0.502	0.635	0.689	0.712	0.809

The diagonal value is the square root of each variable AVE, and the lower triangle is the correlation coefficient between latent variables.

**Table 6:** HTMT discriminant validity*.*

Variables	PEOU	PU	UA	PVE	BI
PEOU	–				
PU	0.712	–			
UA	0.678	0.795	–		
PVE	0.601	0.723	0.789	–	
BI	0.582	0.701	0.765	0.802	–

All values are less than the strict critical value of 0.85, indicating good discriminant validity.

**Table 7:** Model comparisons*.*

Model	*χ*^2^/*df*	CFI	TLI	RMSEA	SRMR	*Δχ^2^*	*Δdf*	*p*
Baseline：PVE First-order single factor	1.84	.991	.987	.036	.024	Reference	—	—
Competing：4 independent factors	4.72	.943	.912	.077	.068	127.41	6	<.001

To test the hypothesized relationships, we estimated a full-mediation baseline structural model alongside a competing partial-mediation nested model for model comparison. The full-mediation model yielded an acceptable fit: $\chi^{2}/df=2.45$, CFI = .938, TLI = .931, RMSEA = .048, SRMR = .041, AIC = 1,892.35, BIC = 2,015.72; the partial-mediation competing model produced fit indices of $\chi^{2}/df=2.39$, CFI = .941, TLI = .934, RMSEA = .047, SRMR = .040, AIC = 1,889.12, BIC = 2,026.45. The chi-square difference test indicated no significant difference between the two models $\Delta \chi^{2}=3.23,\;\Delta df=4,\;(p\;>\;.05)$. Given its lower BIC value and theoretical consistency, the full-mediation model was retained for subsequent hypothesis testing. As shown in Table 8, all direct paths corresponding to H1–H5 were positive and statistically significant. Perceived ease of use was positively associated with perceived usefulness (β=.623),(p<.001), supporting H1. Perceived usefulness was positively related to usage attitude (β=.412),(p<.001), supporting H2. Perceived ease of use was positively associated with usage attitude (β=.356),(p<.001), supporting H3. Usage attitude was positively associated with behavioral intention (β=.689),(p<.001) and perceived viewing-experience enhancement (β=.703),(p<.001), supporting H4 and H5, respectively. Four demographic covariates (gender, age, education, and weekly sports-viewing duration) were included in the structural model. Only weekly sports-viewing duration was positively associated with behavioral intention (β=.102),(p<.05); other demographic variables produced non-significant coefficients, and inclusion of these controls did not alter the significance of hypothesized paths. Robustness analysis was performed by re-estimating the full-mediation structural model without demographic control variables (Supplementary Appendix Table S3). The magnitude and significance of all core direct paths and bootstrap-mediation effects were nearly identical to the baseline model with controls. None of the hypothesis-testing results changed, indicating that the study's substantive conclusions are robust regardless of whether demographic covariates are included.

**Table 8:** Direct path coefficient of structural model*.*

PATH	Standardized coefficient *β*	Standard Error SE	*t*-value	*p*-value	Hypothesis Support
PEOU → PU (H1)	0.623	0.052	11.98	<0.001	Support
PU → UA (H2)	0.412	0.048	8.58	<0.001	Support
PEOU → UA (H3)	0.356	0.045	7.91	<0.001	Support
UA → BI (H4)	0.689	0.051	13.51	<0.001	Support
UA → PVE (H5)	0.703	0.049	14.35	<0.001	Support
control variable：watch_time → BI	0.102	0.041	2.49	<0.05	–

The model controlled for the effects of gender, age, and education level on BI and PVE, all of which were not significant.

Mediating effects of usage attitude were examined in lavaan with 5,000 bootstrap resamples using BCa confidence intervals (Table 9). Beyond the hypothesized simple indirect paths, the model also contains chained sequential indirect effects where perceived ease of use influences outcomes sequentially via perceived usefulness and usage attitude. Table 9 reports all specific indirect effects as well as aggregated total indirect effects for each antecedent. For H6, which hypothesized the mediating role of usage attitude between antecedent variables and behavioral intention, the indirect effect of perceived ease of use on behavioral intention via usage attitude was significant (β=.245), 95% BCa CI [0.189, 0.302]), supporting H6a. The indirect effect of perceived usefulness on behavioral intention through usage attitude was also significant (β=.284), 95% BCa CI [0.221, 0.347]), supporting H6b. Regarding H7, which concerned the mediating mechanism predicting perceived viewing-experience enhancement, perceived ease of use exhibited a significant indirect association with perceived viewing-experience enhancement through usage attitude (β=.250), 95% BCa CI [0.192, 0.308]), supporting H7a. Similarly, the indirect effect of perceived usefulness on perceived viewing-experience enhancement mediated by usage attitude was significant (β=.290), 95% BCa CI [0.226, 0.354]), supporting H7b. Consistent with the full-mediation specification, total effects equaled indirect effects, yielding a mediation proportion of 1.0. This value arises from model constraints rather than effect magnitude, so interpretations focus primarily on indirect coefficient sizes and bootstrap confidence intervals.

**Table 9:** Bootstrap test for specific and total indirect mediation effects.

Mediation Path	Hypothesis	Specific Indirect Effect *β*	Standard Error *SE*	95% BCa Confidence Interval	*p*-value	Hypothesis Support
Outcome: Behavioral Intention (BI)						
PEOU → UA → BI	H6a	0.245	0.029	[0.189, 0.302]	<0.001	Support
PEOU → PU → UA → BI	Chained sequential indirect	0.174	0.024	[0.131, 0.222]	<0.001	-
Total indirect effect PEOU → BI	-	0.419	0.038	[0.348, 0.496]	<0.001	-
PU → UA → BI	H6b	0.284	0.032	[0.221, 0.347]	<0.001	Support
Total indirect effect PU → BI	-	0.284	0.032	[0.221, 0.347]	<0.001	-
Outcome: Perceived viewing-experience enhancement (PVE)						
PEOU → UA → PVE	H7a	0.250	0.030	[0.192, 0.308]	<0.001	Support
PEOU → PU → UA → PVE	Chained sequential indirect	0.178	0.025	[0.134, 0.228]	<0.001	-
Total indirect effect PEOU → PVE	-	0.428	0.039	[0.354, 0.507]	<0.001	-
PU → UA → PVE	H7b	0.290	0.033	[0.226, 0.354]	<0.001	Support
Total indirect effect PU → PVE	-	0.290	0.033	[0.226, 0.354]	<0.001	-

Bootstrap sampling 5,000 times, using BCa confidence interval estimation.

## 5 Discussion

Drawing on the Technology Acceptance Model (TAM), this study examined statistical associations among audience technology perceptions, usage attitude, behavioral intention, and perceived viewing experience enhancement (PVE) in the context of AI-based sports broadcasting services. Unlike many prior TAM-informed sport media studies that treat behavioral intention as the sole outcome, the present work incorporated PVE—an individual level subjective experiential outcome—alongside the canonical behavioral intention construct.

Consistent with classic TAM findings from general digital technology contexts (13, 39), perceived ease of use was positively associated with perceived usefulness. Both perceived ease of use and perceived usefulness showed positive statistical associations with usage attitude. These patterns align with meta-analytic syntheses of TAM, which indicate that perceptual beliefs about technology tend to correlate with users' evaluative attitudes toward digital services (20). In the specific setting of AI-based sports broadcasting, the results suggest that audiences' judgments about operational simplicity and functional value are closely linked to how they evaluate AI-enhanced viewing features.

Usage attitude was positively associated with both behavioral intention and PVE. This pattern aligns with cross-disciplinary research evidence that documents the association between attitudes and outcomes across various digital media environments (22, 24). However, most earlier empirical work has centered on behavioral or usage intention indicators, while subjective experiential gains such as PVE have rarely been incorporated within TAM-based sport broadcasting research. The present findings help address this gap by showing that favorable usage attitudes are statistically linked not only to willingness for continued engagement but also to self-reported improvements in individual level match comprehension, emotional immersion, and viewing satisfaction.

Consistent with the *a priori* full mediation specification, bootstrap-based mediation tests indicated that usage attitude functions as a mediating construct linking the perceptual predictors (perceived ease of use and perceived usefulness) to the two outcome variables (BI and PVE). Nested model comparison showed that adding direct paths from the perceptual predictors to the outcomes did not yield meaningful improvement in model fit. This underscores the central statistical role of usage attitude within the present sample: technology-related perceptions correlate with user-level outcomes primarily through users' overall evaluative stance toward AI-enhanced broadcasting features. The finding echoes attitude-centered extensions of TAM reported in information systems research (26, 29), yet it has been less frequently examined in sports media consumption contexts characterized by experiential viewing goals.

These observed associational patterns cannot be fully understood only by referencing general purpose TAM findings; they also reflect unique characteristics of AI-based sports broadcasting as a hedonic, experience centered media scenario. Unlike utilitarian traditional digital system contexts (such as office software or information management platforms), where users primarily pursue task completion, audiences consume AI-based sports broadcasting for recreational, affective, and experiential rewards. In this scenario, perceived ease of use and perceived usefulness do not serve purely instrumental purposes: easy to operate AI functions remove distractions from match watching, while useful AI features such as intelligent commentary and personalized highlights directly enrich the live viewing experience itself, rather than merely improving work related task efficiency. This experiential nature helps explain why usage attitude exhibits a strong correlation with perceived viewing experience enhancement (PVE). For sports viewing audiences, usage attitude is not merely a cold cognitive evaluation of technical performance; it captures holistic affective appraisal tied to subjective enjoyment during match consumption. When audiences form positive overall attitudes toward AI-enhanced broadcasting features, they are more inclined to subjectively register improvements in match comprehension, emotional immersion and interactivity that constitute PVE. By contrast, in many conventional utilitarian technology settings, attitude tends to predict future behavioral adoption more strongly, while subjective experiential quality remains secondary. Even so, alternative plausible interpretations should be acknowledged. The strong attitude—PVE association may partly stem from affective response spillover: general positive affect toward sports events themselves could simultaneously lift both attitude toward AI functions and self-reported viewing experience. In addition, common-method variance from self-report surveys may inflate the magnitude of this correlation, although our diagnostic tests suggested common-method bias was not severe within this dataset.

### 5.1 Theoretical implications

This study offers modest, context specific insights for technology acceptance scholarship in sport media domains rather than a fundamental theoretical expansion of the TAM framework.

First, the results illustrate that the core nomological associations of TAM can be observed within the entertainment-oriented consumption context of AI-based sports broadcasting. Perceived ease of use and perceived usefulness maintain meaningful statistical links to usage attitude, which in turn correlates with user-level outcomes. This adds further empirical evidence for the applicability of TAM-derived reasoning in non-Western sport media audience samples (12, 18).

Second, by integrating perceived viewing experience enhancement as an additional individual centered experiential outcome alongside behavioral intention, the study demonstrates that attitudinal evaluations of AI sports services correlate not only with behavioral willingness but also with self-reported viewing experience gains. In entertainment-oriented media consumption settings, an exclusive focus on behavioral intention metrics may overlook subject-centered experiential responses that matter for audience reception. This represents a context-oriented adjustment for sport media research rather than a broad theoretical breakthrough for TAM itself. This dual focus moves the application of TAM beyond the prediction of adoption intention alone and allows the model to capture the experiential consequences that are central to hedonic media consumption. Rather than treating PVE as a simple additional dependent variable, the present specification theoretically links technology perceptions to both behavioral and experiential outcomes through the common mediating mechanism of usage attitude, thereby offering a more complete account of audience responses in AI-enhanced sports broadcasting.

Third, the mediation results reinforce the psychological standing of usage attitude. In this sample, technology perceptions are statistically related to outcomes predominantly via usage attitude. The finding is congruent with attitude-focused TAM extensions in general digital technology research (26) and provides sport-specific empirical evidence for the attitudinal mediation mechanism in experiential media use scenarios.

Notably, this strong mediating role of usage attitude should be interpreted within this hedonic sports viewing context and cannot be automatically generalized to utilitarian AI technology scenarios. We also acknowledge competing potential explanations: pre-existing sports involvement or event derived positive emotion might amplify both attitude and experiential perceptions, even independent of AI-feature quality. Such unmeasured confounding variables constitute one boundary for interpreting the present correlational results.

### 5.2 Practical implications

First, perceived ease of use remains a relevant perceptual correlate of audience evaluations. Platform and product developers may prioritize intuitive interfaces and low complexity interaction designs for AI-enabled sports viewing functions. Reducing unnecessary operational burdens may help foster more favorable user evaluations of these features.

Second, perceived usefulness exhibits meaningful statistical associations with usage attitude. Practitioners could prioritize functional features that deliver tangible viewing-related benefits, such as supporting match comprehension, providing personalized content recommendations, and supplying relevant event-related information. Features that do not yield observable viewing-related gains may be less effective in cultivating positive user attitudes, even when the underlying AI technology is sophisticated.

Third, usage attitude correlates positively with both behavioral intention and perceived viewing experience enhancement. Cultivating favorable evaluative attitudes toward AI-based sports broadcasting services appears relevant for sustaining audience engagement and supporting self-reported subjective viewing experience gains. Product iterations may therefore consider how functional design shapes users' overall attitudes toward AI-driven viewing functions, beyond purely technical performance indicators.

### 5.3 Research limitations and future directions

Several limitations should be noted.

First, the study relies on cross-sectional survey data. All reported results reflect statistical associations among variables and cannot establish causal ordering. Although the structural model follows theoretically derived directional hypotheses, cross-sectional data cannot rule out alternative reciprocal relationships. Longitudinal designs would help clarify temporal sequences among audience perceptions, attitudes, and viewing-related responses.

Second, all measures depend on participants' subjective self-reports, which carry inherent risks of common method variance and social desirability bias, even though diagnostic tests suggested that common method bias was not severe in this dataset. Future investigations could combine self-report responses with objective behavioral records where feasible.

Third, convenience sampling was used, and the sample contains a relatively high proportion of young respondents (52.5% aged 18–24). Younger audiences typically possess higher digital literacy and greater openness toward emerging AI-enhanced media services. Consequently, the observed correlational patterns cannot be readily generalized to the broader population of Chinese sports audiences; older demographic subgroups may display different relationships. Stratified sampling covering wider age ranges would help test the robustness of these associations.

Fourth, this study only screened respondents based on self-reported general usage experience with AI-enhanced sports broadcasting functions. Detailed information on specific platforms, usage frequency, and the recency of last usage was not collected, which prevents finer grained subgroup comparisons across different user profiles.

## 6 Conclusion

In summary, the present study investigates the correlational relationships among perceived ease of use, perceived usefulness, usage attitude, behavioral intention, and perceived viewing-experience enhancement in the context of AI-based sports broadcasting services. The findings show that perceived ease of use and perceived usefulness are positively associated with usage attitude, and that usage attitude is positively associated with both behavioral intention and perceived viewing-experience enhancement. Usage attitude functions as a full mediator linking the two perceptual predictors to the two outcome variables.

This work extends TAM-related empirical evidence to the setting of AI-based sports broadcasting and illustrates the value of incorporating an individual-level experiential outcome alongside the conventional behavioral-intention construct. Practitioners may benefit from prioritizing ease of use and functional usefulness in the design of AI broadcasting features, and from fostering positive user attitudes, so as to support both continued engagement and enhanced viewing experiences.

## Data Availability

Due to privacy and ethical restrictions imposed by participant informed consent, the raw data underlying the conclusions of this article are not publicly available. Please contact the author to request access to the dataset. Liuxin Li, 2595414211@qq.com.
