# Supplementary material for: Perceived viewing-experience enhancement and audience acceptance: applying TAM to AI-based sports broadcasting services
Source: Front Sports Act Living. 2026 Oct 16;8:1945033. doi: 10.3389/fspor.2026.1945033 (PMC13721283; doi:10.3389/fspor.2026.1945033)
Supplement: Supplementary file 1 [file Supplementaryfile1.docx]

**Appendix**

**Table S2‑1.** *Baseline CFA vs Latent Common‑Method‑Factor augmented model fit comparison.*

| Model | χ2/df | CFI | TLI | RMSEA | SRMR |
| --- | --- | --- | --- | --- | --- |
| Baseline five‑factor CFA | 2.31 | 0.942 | 0.935 | 0.046 | 0.038 |
| Latent common‑method‑factor augmented model | 2.17 | 0.948 | 0.941 | 0.043 | 0.041 |

**Table S2‑2.** *Changes in standardized substantive indicator loadings after adding common‑method factor.*

| Item | Loading (baseline CFA) | Loading (CMF‑augmented model) | Absolute change |
| --- | --- | --- | --- |
| PEOU1 | 0.762 | 0.741 | 0.021 |
| PEOU2 | 0.774 | 0.748 | 0.026 |
| PEOU3 | 0.751 | 0.727 | 0.024 |
| PEOU4 | 0.780 | 0.752 | 0.028 |
| PU1 | 0.771 | 0.744 | 0.027 |
| PU2 | 0.783 | 0.751 | 0.032 |
| PU3 | 0.790 | 0.756 | 0.034 |
| PU4 | 0.787 | 0.753 | 0.034 |
| UA1 | 0.782 | 0.746 | 0.036 |
| UA2 | 0.791 | 0.754 | 0.037 |
| UA3 | 0.788 | 0.749 | 0.039 |
| UA4 | 0.801 | 0.760 | 0.041 |
| PVE1 | 0.785 | 0.743 | 0.042 |
| PVE2 | 0.794 | 0.750 | 0.044 |
| PVE3 | 0.798 | 0.752 | 0.046 |
| PVE4 | 0.810 | 0.763 | 0.047 |
| BI1 | 0.792 | 0.747 | 0.045 |
| BI2 | 0.804 | 0.754 | 0.050 |
| BI3 | 0.797 | 0.745 | 0.052 |
| BI4 | 0.812 | 0.734 | 0.078 |

**Table S3.** *Robustness check: comparison of model with / without demographic control variables.*

| Path | Model with controlsβ | p‑value | Model without controlsβ | p‑value |
| --- | --- | --- | --- | --- |
| PEOU → PU (H1) | 0.623 | <0.001 | 0.627 | <0.001 |
| PU → UA (H2) | 0.412 | <0.001 | 0.416 | <0.001 |
| PEOU → UA (H3) | 0.356 | <0.001 | 0.351 | <0.001 |
| UA → BI (H4) | 0.689 | <0.001 | 0.692 | <0.001 |
| UA → PVE (H5) | 0.703 | <0.001 | 0.705 | <0.001 |

**Supplementary references**

[40]Achen, R.M., Lebel, K., & Clavio, G. (2017). What customers want: Defining engagement on

social media in sport. *Global Sport Business Journal, 5 (3)*, 1.

https://scholarworks.sfasu.edu/gsbj/vol5/iss3/1

[41]Li, F., Ren, H., & Yoon, J. (2026). Artificial Intelligence–Enhanced Analytics in Sport

Communication: Advancing Environmental Sustainability Through Stakeholder-Centered

Strategies. *International Journal of Sport Communication, 1 (aop)*, 1-12.

[https://doi.org/10.1123/ijsc.2025-0196](https://doi.org/10.1123/ijsc.2025-0196)

[42]McQuail, D. (2010). McQuail’s mass communication theory . *Sage publications.*

[43]Min, J., Xie, Q., & Liu, Y. (2025). Impact of Artificial Intelligence on Spectator Viewing

Behavior in Sports Events: Mediating Role of Viewing Motivation and Moderating Role of Player

Identification. *Behavioral Sciences, 15(12)*, 1702. <https://doi.org/10.3390/bs15121702>

[44]Al-Emran, M., Mezhuyev, V., & Kamaludin, A. (2018). Technology Acceptance Model in

M-learning context: A systematic review. *Computers & Education, 125,* 389-412.

[https://doi.org/10.1016/j.compedu.2018.06.008](https://doi.org/10.1016/j.compedu.2018.06.008)

[45]Haleem, M.M.A., & Alnajjar, H.T.(2024). The Role of Virtual Social Presence in Arab

Audience Acceptance of AI in Media Practices: Insights from the Technology Acceptance Model.

A*rab Media and Society, 2024(37)*, 1-28. https://doi.org/10.70090/MH24RVSP

[46]Venkatesh, V., Thong, J.Y., & Xu, X. (2012). Consumer acceptance and use of information

technology: Extending the Unified Theory of Acceptance and Use of Technology1. *MIS quarterly,*

*36 (1),* 157-178. [https://doi.org/10.2307/41410412](https://doi.org/10.2307/41410412)

[47]Wang, X. F., Wang, Z. Z., & Yan, Y. R. (2021). Application of artificial intelligence and

transformation of sports communication. *Journal of Shanghai University of Sport, 45(2)*, 57‑64.

[https://doi.org/10.16099/j.sus.2021.02.007](https://link.wtturl.cn/?target=https://doi.org/10.16099/j.sus.2021.02.007&scene=im&aid=582478&lang=zh)

[48]Won, D., Chiu, W., & Byun, H. (2023). Factors influencing consumer use of a sport-branded

app: the technology acceptance model integrating app quality and perceived enjoyment. A*sia*

*Pacific Journal of Marketing and Logistics, 35(5),* 1112-1133.

[https://doi.org/10.1108/APJML-09-2021-0709](https://doi.org/10.1108/APJML-09-2021-0709)

[49]Muzhingi, S. A., & Appelman, A. (2025). Ethics’ role in AI adoption: Extending the

Technology Acceptance Model. *Journal of Communication Technology, 7(3)*, 51‑69.

[https://doi.org/10.51548/joctec.7.3.2025.03](https://link.wtturl.cn/?target=https://doi.org/10.51548/joctec.7.3.2025.03&scene=im&aid=582478&lang=zh)

[50]Oc, Y. & Toker, A. (2022). An acceptance model for sports technologies: the effects of sports

motivation, sports type and context-aware characteristics. *International Journal of Sports*

*Marketing and Sponsorship, 23(4)*, pp. 785-803. doi: 10.1108/ijsms-03-2021-0060

[51]King, W. R., & He, J. (2006). A meta‑analysis of the technology acceptance model.

*Information & Management, 43(6),* 740‑755. [https://doi.org/10.1016/j.im.2006.05.003](https://link.wtturl.cn/?target=https://doi.org/10.1016/j.im.2006.05.003&scene=im&aid=582478&lang=zh)

[52]Koppel, S., Logan, D.B., Zou, X., Kaviani, F., McDonald, H., Hair Jr, J.F., ... & Charlton, J.L.

(2024). Factors influencing behavioral intentions to use conditionally automated vehicles. *Journal*

*of Safety Research, 91*, 423-430. [https://doi.org/10.1016/j.jsr.2024.10.006](https://doi.org/10.1016/j.jsr.2024.10.006)
